# Frontal Electroencephalogram Alpha Asymmetry during Mental Stress Related to Workplace Noise

**DOI:** 10.3390/s21061968

**Published:** 2021-03-11

**Authors:** Emad Alyan, Naufal M. Saad, Nidal Kamel, Mohd Zuki Yusoff, Mohd Azman Zakariya, Mohammad Abdul Rahman, Christophe Guillet, Frederic Merienne

**Affiliations:** 1 Centre for Intelligent Signal and Imaging Research (CISIR), Department of Electrical and Electronic Engineering, University Teknologi PETRONAS, Seri Iskandar 32610, Perak, Malaysia; emadalyan@gmail.com (E.A.); naufal_saad@utp.edu.my (N.M.S.); nidalkamel2@hotmail.com (N.K.); 2 Department of Electrical and Electronic Engineering, University Teknologi PETRONAS, Seri Iskandar 32610, Perak, Malaysia; mazman_zakariya@utp.edu.my; 3 Faculty of Medicine, University Kuala Lumpur Royal College of Medicine Perak, Ipoh 30450, Perak, Malaysia; mohammad@unikl.edu.my; 4 Institut Image 2 rue T. Dumorey, LISPEN EA 7515, Universite de Bourgogne, UBFC, 71100 Chalon-sur-Saone, France; christophe.guillet@u-bourgogne.fr; 5 Arts et Metiers Institute of Technology, LISPEN, HESAM Université, UBFC, F-71100 Chalon-sur-Saône, France; frederic.merienne@ensam.eu

**Keywords:** electroencephalogram (EEG), noise stress, EEG alpha-asymmetry, prefrontal cortex, salivary alpha-amylase

## Abstract

This study aims to investigate the effects of workplace noise on neural activity and alpha asymmetries of the prefrontal cortex (PFC) during mental stress conditions. Workplace noise exposure is a pervasive environmental pollutant and is negatively linked to cognitive effects and selective attention. Generally, the stress theory is assumed to underlie the impact of noise on health. Evidence for the impacts of workplace noise on mental stress is lacking. Fifteen healthy volunteer subjects performed the Montreal imaging stress task in quiet and noisy workplaces while their brain activity was recorded using electroencephalography. The salivary alpha-amylase (sAA) was measured before and immediately after each tested workplace to evaluate the stress level. The results showed a decrease in alpha rhythms, or an increase in cortical activity, of the PFC for all participants at the noisy workplace. Further analysis of alpha asymmetry revealed a greater significant relative right frontal activation of the noisy workplace group at electrode pairs F4-F3 but not F8-F7. Furthermore, a significant increase in sAA activity was observed in all participants at the noisy workplace, demonstrating the presence of stress. The findings provide critical information on the effects of workplace noise-related stress that might be neglected during mental stress evaluations.

## 1. Introduction

In the modern age, noise is increasingly becoming the most prevalent pollutant in the workplace environment. The use of different workplace tools and appliances can increase the noise pollution/stress to the individuals working in their environment [1]. In addition, various forms of noise stressors typically encountered in urban environments include workplace noise (building sites, busy office, workplace tools), transport noise (buses, cars, trains), and social and home noise (people talking, busy cafes, continuously running televisions). It has been estimated that half of the population in the UK lives at levels of environmental noise that surpass the standards of the World Health Organization (WHO) [2]. Noise has been described as an unwanted sound that can act as both a physical and a psychological stimulus [3]. It consists of extremely irritating, distracting, or repetitive sounds, which disrupt the ability to concentrate. It has been recognized by the World occupational Safety and Health Organizations as a psychobiological stressor because of its effect on the central nervous system and general well-being, causing physiological, psychological, and behavioral changes in healthy subjects [4,5,6]. Noise and its potential implications on workers and well-being are of great concern in workplace settings where noise levels often exceed 70 decibels (dB), influencing the hypothalamic-pituitary-adrenal (HPA) axis and the limbic system.

People spend much of their time in the working environment, frequently overburdened by heavy workloads, time pressure, and other physical stressors, factors that tend to increase stress levels. In real life, workplace stressors can be found individually or combined in various forms, including psychosocial and environmental stressors, which have adverse effects on employees’ health and wellbeing [7]. Psychosocial stressors such as time constraints, high workload, and job characteristics put employees under tremendous psychological and social strains, which negatively affect their performance [8], precision [9], and decisions [10]. Besides, environmental stressors such as noise have been reported to impair cognitive performance by disturbing the decision-making process and attention selection [11]. According to the literature, noise stress has a detrimental effect on health, working memory, attention, and job satisfaction [12,13], which varies according to the task’s difficulty. For example, the noise does not affect simple tasks but plays a factor in more complex tasks [14]. Further, noise is capable of disturbing a worker’s performance not only by chronic exposure but also by acute high-volume exposure [15,16]. Thus, a reduction in workplace noise could help to reduce the negative impacts of work stress [17].

The brain plays a critical role in regulating and responding to stress, particularly during the potentially threatening situation, and also controls individuals’ behavioral and physiological responses [18]. By stimulating the reticular activating system (RAS), noise impacts neural impulses from the RAS to the cerebral cortex, and this affects cognitive behavior and deteriorates performance at the workplace [19]. Generally, stressors stimulate the sympathetic–adrenal–medullary (SAM) axis, resulting in increased activity of salivary alpha-amylase (sAA) [20]. sAA is a salivary enzyme involved in the metabolism of carbohydrates and starches. It has emerged as a valid and reliable marker for sympathetic activity [8,21,22]. The sAA is responsive to stress stimuli and substantially increased in response to them. Evidence indicates that measurements of sAA are associated with norepinephrine variations, prompting researchers to use sAA as an indirect measure for the activation of the SAM axis [23,24]. According to the literature, the sAA level is rapidly increased than the salivary cortisol level, which takes more than 10 min to react.

During chronic stress induced by persistent exposure to noise, the activation of the SAM system alerts the cortex to the action of corticotropic hormone-producing and also stimulates the cortex directly. Note that chronic stress induces significant changes in the sympathetic neural system related to behavioral repression. Because of the complex neural links between the prefrontal cortex (PFC) and limbic systems, noise stress may have a broader negative impact on cognition and emotion. Noise stress has been documented to impair cognitive function via dopaminergic mechanisms, primarily in the PFC, as was apparent in several studies. Despite the homeostasis responses at the amygdala, the elevated level of catecholamine following or during noise stress rapidly impairs cognitive function [25]. The PFC can change the reward circuit due to its influence over dopamine release [26,27]. The PFC is sufficiently resilient to balance dopamine levels in the limbic system. This is accomplished by the PFC using feedback information obtained from the limbic system.

The development of neuroimaging modalities has advanced our understanding of brain function during the interaction between various stress contributors. Nonetheless, few studies investigated the interaction of cognitive and physical stressors [14]. Mehta et al., for example, reported that PFC interference can affect motor performance during tasks that require both cognitive and physical processing [28]. Additionally, consolidated physical and mental stress showed a substantial increase in pulse and systolic blood pressures during hypoxic conditions, while separated mental stress has not affected under the same conditions [29]. Furthermore, lateralized neocortex activation can be efficiently evaluated using frontal alpha-asymmetry (FAA) measures of electroencephalogram (EEG). Alpha asymmetry can serve as an effective moderator for assessing stress-related disorders in real-life contexts [30]. The neurophysiological research demonstrates that the left brain is more active in positive emotions and approach-related activities, and the right side of the brain is more involved in negative emotional regulation and social withdrawal behaviors [31,32]. More specifically, alpha power is inversely associated with the cortical activity [33], and an increase in alpha power is correlated with the functional suppression of subcortical areas that respond to irrelevant information to the task at hand. However, previous studies reported relaxation states with relatively greater alpha activity in contrast to stressful conditions [34,35,36].

The objective of this research is to examine the effects of workplace noise and its interaction with psychosocial stressors on the FAA scores and sAA levels. Two workplace environments (quiet versus noisy) are designed and examined under conditions of mental stress using the Montreal Imaging Stress Task (MIST), in which MIST reported to increase the levels of cortisol and differentially affect brain activity [37].

## 2. Materials and Methods

### 2.1. Participants

The study was announced through a recruitment notice advertisement to include healthy non-smoking volunteers being right-handed with no history of psychiatric conditions or neurological illnesses and with normal or corrected visual acuity. Subjects who met all selection criteria were then provided with detailed information about the purpose and process of the study. Every participant has given informed consent to their involvement before the experiment. After providing signed consent, participants (n = 18; males; average age 27.2 ± 2.8 years) who met all eligibility criteria were randomly recruited for the experiment. Data of volunteers who failed to follow instructions or showed low data quality due to their excessive head motion were excluded from the analysis (n = 3). Therefore, data from fifteen right-handed male participants were included in the final analysis (n = 15). The study protocol was approved by the ethics committee (UniKLRCMP/MREC/2020/132) of Universiti Kuala Lumpur Royal College of Medicine Perak (UniKL RCMP). All procedures were conducted following the approved regulations and guidelines.

### 2.2. Experimental Procedure

The experiment was designed to consider two types of workplaces, quiet and noisy. The noisy workplace involved noise sources that were presented by audio files consisted of workplace realistic individual or combined sounds such as paper printing, moving furniture, telephone ringing, and others. These noise stressors were non-repeatable and randomly presented to participants, and their levels varied between 64.4 and 76.8 dB, with a mean of 70.6 dB. However, both workplaces were associated with psychosocial stressors sourced from the MIST [37], which consisted of the mental arithmetic task (MAT) along with time constraints and social assessment threats. The MAT included three random integers ranging from 0 to 99, with random operators such as plus ‘+’ and minus ‘−’ (example: 28 − 35 + 9). The MIST was selected in this study owing to its capability to induce reliable stress engaging the HPA axis [37,38,39].

**Figure 1:**
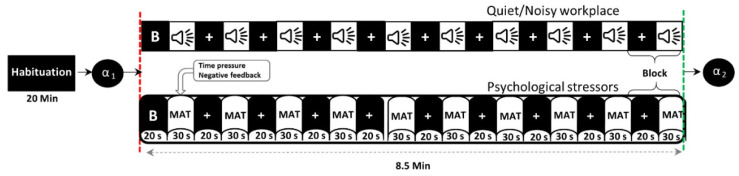
Timeline of the experimental protocol (B = baseline, α = alpha-amylase, MAT = mental arithmetic task).

Figure 1 shows the timeline of the experiment in a block design, in which participants performed the MIST in both quiet and noisy workplaces that were randomly assigned to minimize the learning effect. Before the experiment (habituation period), participants were given a brief introduction to allow them to become habituated to their surroundings. In this time, participants were also trained in solving sample questions from the real MAT as fast as possible without time constraints, noise stressors, or social assessment threats to estimate the average time taken per problem. During the experiment, each participant completed the MIST with overlapping environmental noises and psychosocial stressors for ten blocks, each for 30 s of the task and 20 s of rest. To further increase participants’ stress, 90% of the average time taken during the training session was defined as a time constraint, and 85% of the actual average performance was displayed on the screen during the experiment session. Furthermore, the time constraint has been adaptively reduced or increased by 10% after three consecutive correct or incorrect answers, respectively. All participants were encouraged to calm and concentrate on the fixation cross that appeared on the screen during baseline and rest conditions. The social assessment threats include feedback, such as incorrect, correct, or timeout, shown on the screen according to the participant’s response within the specified time limit. The activity of salivary-alpha amylase was measured before and immediately after each experiment to validate participants’ stress levels.

### 2.3. Physiological Measurements

A total of two saliva samples in each session was taken from all participants to measure their stress level. We collected the samples using COCORO Meter (Nipro Co., Osaka, Japan) [40,41]. Each participant provided one sample before the session as a baseline, and a second sample, immediately after completion of the session. For each collection of saliva samples, participants were instructed to insert a new strip into their mouth for approximately 30s. We then placed the strip into the COCORO Meter to get the stress levels by measuring the enzyme amylase level in saliva.

### 2.4. EEG Data Acquisition and Pre-Processing

**Figure 2:**
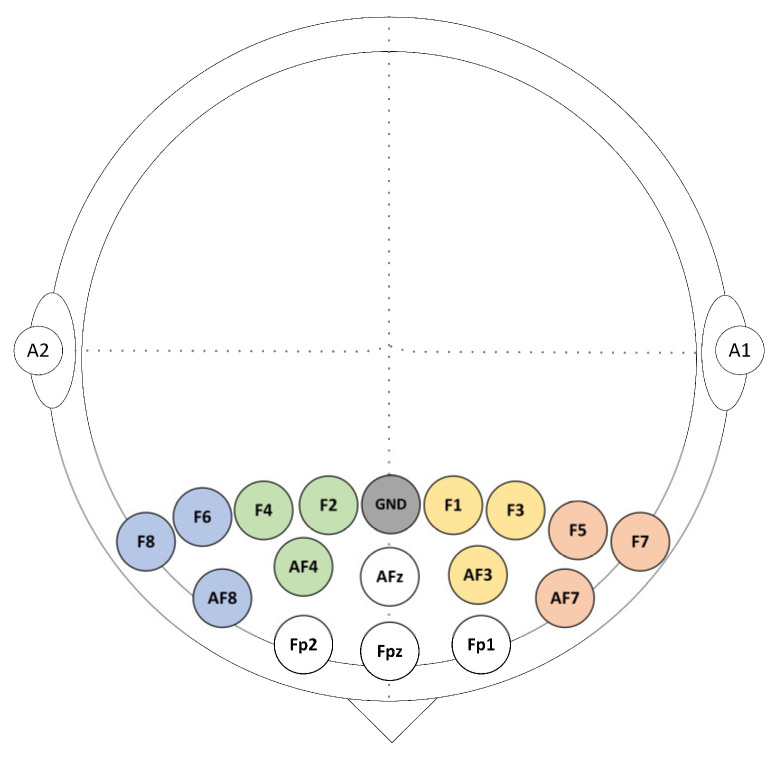
Sixteen EEG selected channels were arranged above the prefrontal cortex (PFC).

EEG data were acquired from a total of 16 gold electrodes at a rate of 256 Hz using BrainMaster Discovery amplifier (BrainMaster Technologies Inc., Bedford, USA). These electrodes were placed on the participants’ frontal cortex at the following locations: Fz, Fp1, Fp2, AFz, AF3, AF4, AF7, AF8, F1, F2, F3, F4, F5, F6, F7, and F8 according to the 10/10 system. All electrodes were grounded at the Fz and referenced to the linked earlobes as highlighted in Figure 2. The electrodes’ impedances were kept underneath 5 kΩ to ensure high-quality EEG recordings. The event trigger signals obtained were sent from the parallel port of the computer accessed by MATLAB to the EEG amplifier and recorded on an event channel to synchronize the presentation of tasks and the recordings of EEG data.

The raw EEG data collected was preprocessed using the EEGLAB toolbox [42] for MATLAB. This includes filtering unwanted frequencies and referencing using the grand average reference algorithm implemented in EEGLAB routines. EEG epochs were obtained using a time frame of 50 s (20 s before the onset and ended 30 s after) and were divided according to the workplace environment, quiet or noisy. Noisy EEG waveforms were excluded through a visual examination. The EEG data were divided into independent components using the EEGLABrunica function. Components reflecting ocular movements and muscular contractions were omitted from the neurological data by visual inspection.

### 2.5. EEG Data Analysis

All artifact-free data that was 30 s segments for stress tasks was subjected to a Fast Fourier transform (FFT) with 50% overlapping and a Hanning window to avoid discontinuity errors. The spectral power (microvolt squared) was estimated, which was then converted to a power density function (microvolt-squared / hertz) as a measure of mean spectral power in the alpha frequency range (8–13 Hz) across the epochs within each workplace condition. A natural-log-transformation was implemented to all values of power density to normalize the distribution. Our study focused on F4, F3, F8, and F7 regions, which are commonly used in the literature of frontal alpha asymmetry [43,44,45]. EEG FAA scores were obtained by subtracting natural-log-transformed alpha power of the right frontal electrodes from those on the left (example: F4-F3, F8-F7) [43,44]. The FAA formula is given as:

(1) $$\mathrm{FAA}=ln\;\left(\alpha_{Rchannel}\right)\;-ln\;\left(\alpha_{Lchannel}\right)$$

where *α_Rchannel_* and *α_Lchannel_* indicate the alpha power in the right and left PFC, respectively. As FAA output, positive scores contribute to higher alpha power of the right PFC (or decreased right cortical activity), while negative scores contribute to higher alpha power of the left PFC (or decreased left cortical activity). For the alpha asymmetry analysis, four clusters of electrode channels corresponding to the dorsolateral prefrontal cortex (DLPFC) were formed. The clusters were distributed as F4 (F2, F4, and AF4), F8 (F6, F8, and AF8), F3 (F1, F3, and AF3), and F7 (F5, F7, and AF7).

### 2.6. Statistical Analysis

Statistical analyses were carried out with MATLAB and the Statistical Package for Social Sciences (SPSS) version 25.0 (SPSS Inc., Chicago, IL, USA). Salivary alpha-amylase values were analyzed using a two-way analysis of variance (ANOVA) with Bonferroni’s post hoc tests comprising the within-subjects factors workplace (quiet, noisy) and time (before, immediately after), and their interaction effects. One-way ANOVAs were used to evaluate the effects of stress on alpha power group differences. Effects of stress on FAA scores between quiet and noisy workplaces were analyzed using paired sample *t*-tests as well as using a two-way ANOVA comprising the within-subjects factors workplace (quiet, noisy) and location (F4-F3, F8-F7). All parametric statistics were carried out after confirming the normal distribution. Bonferroni corrections were performed to adjust for multiple comparisons.

## 3. Results

### 3.1. Behavioral and Physiological Responses

#### 3.1.1. sAA Stress Responses

**Figure 3:**
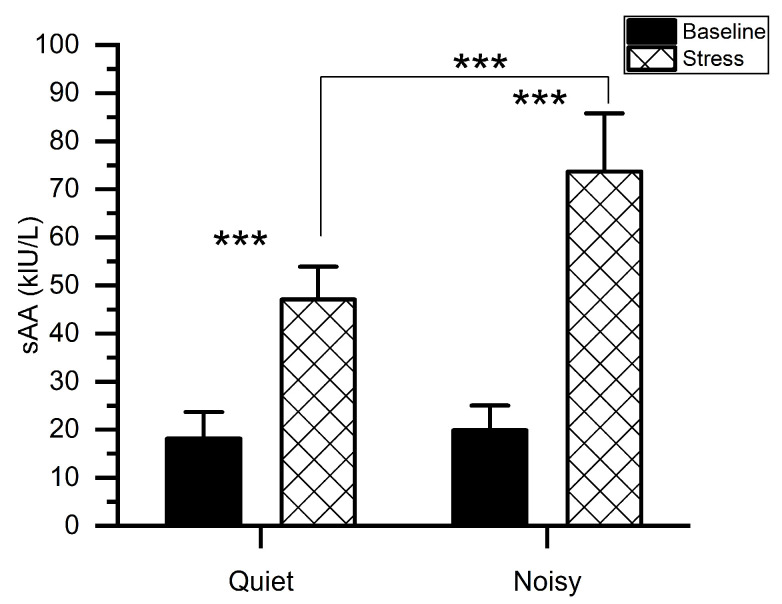
Mean activity of salivary alpha-amylase for stress and baseline at quiet and noisy workplaces. The error bars indicate the standard deviations and the stars indicate the significant differences (*** *p* < 0.001).

Figure 3 shows the mean levels of sAA along with the measurements taken before (baseline) and immediately after each experiment. We conducted a two-way ANOVA to examine the change in sAA levels particularly associated with the MIST in quiet and noisy workplaces. A workplace*time (before and immediately after the experiment) with subjects ANOVA on sAA scores exhibited significant main effects of workplace (*F*(1, 56) = 48.006, *p* < 0.001, *η_p_*^2^ = 0.462), TIME (*F*(1, 56) = 409.316, *p* < 0.001, *η_p_*^2^ = 0.880) as well as a significant workplace*time interaction (*F*(1,56) = 36.977, *p* < 0.001, *η_p_*^2^ = 0.398). Post-hoc pairwise comparisons showed that when subjects were exposed to environmental noises during stress conditions, higher sAA levels were recorded (mean (*M*) = 73.667, standard deviation (*SD*) = 12.128) as compared to the quiet workplace(*M* = 47.067, *SD* = 6.829). There was no difference in sAA values (*t*(1, 14) = 1.193, *p* = 0.253) between quiet (*M* = 18.133, *SD* = 5.527) and noisy (*M* = 19.866, *SD* = 5.153) workplace groups at baseline.

#### 3.1.2. MIST Performance

**Figure 4:**
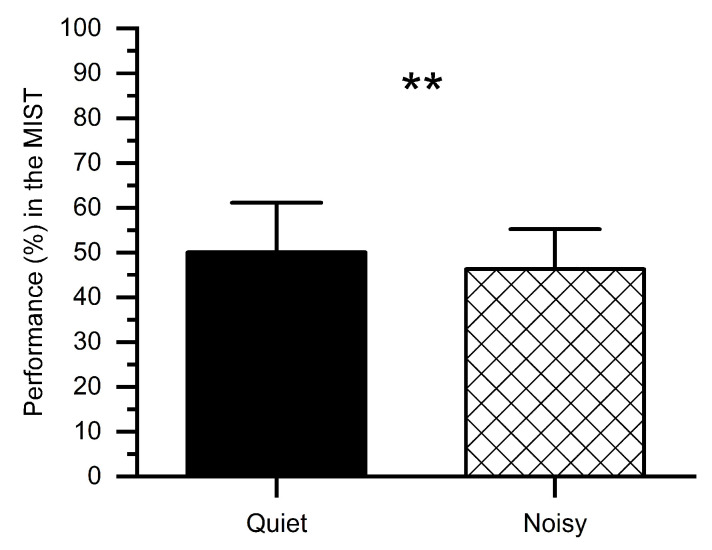
Percent performance in the MIST under stress conditions at quiet and noisy workplaces. Error bars indicate the standard deviations and stars (**) indicate significant differences ** *p* < 0.01.

We computed percent correct performance (number of correct answers) for quiet and noisy workplace groups, as shown in Figure 4. Subjects performed worse in the noisy workplace (*M* = 46.32%, *SD* = 9.78%) as compared to the quiet workplace (*M* = 50.05%, *SD* = 7.91%). The *t*-test revealed that, in the noisy workplace, the performance was significantly worse (*t*(1, 14) = 2.76, *p* < 0.01) in comparison with the quiet workplace, which confirms that a noisy workplace affects people’s performance.

### 3.2. Effects of Stress on Alpha Power and FAA

#### 3.2.1. Alpha Power

**Figure 5:**
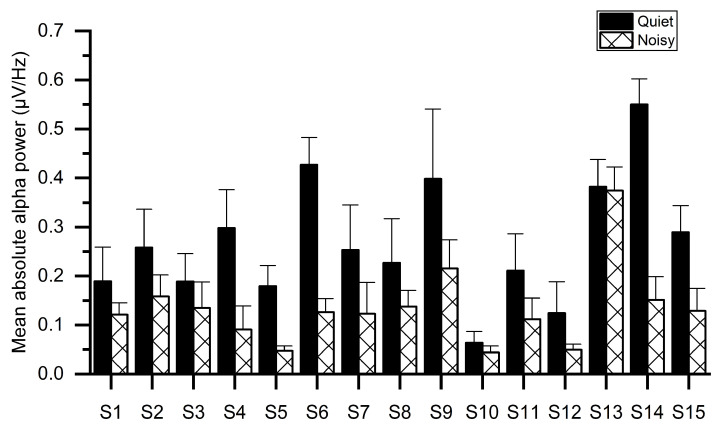
Average absolute alpha power for each subject while working at quiet and noisy workplaces.

Figure 5 illustrates the mean absolute alpha power of all subjects for both workplaces, indicating that the noisy workplace group exhibits lower alpha power as compared with the quiet workplace group. The reduction in alpha power may be owing to the stress as confirmed by sAA levels in Figure 3. One-way ANOVA tests showed significant group differences of absolute alpha power using the mean values of electrodes. The results of ANOVA indicated that the absolute alpha power in the quiet workplace group (*M* = 0.283, *SD* = 0.131) was significantly larger (*F*(1, 29) = 13.641, *p* < 0.001) than in the noisy workplace group (*M* = 0.137, *SD* = 0.08).

#### 3.2.2. FAA

**Figure 6:**
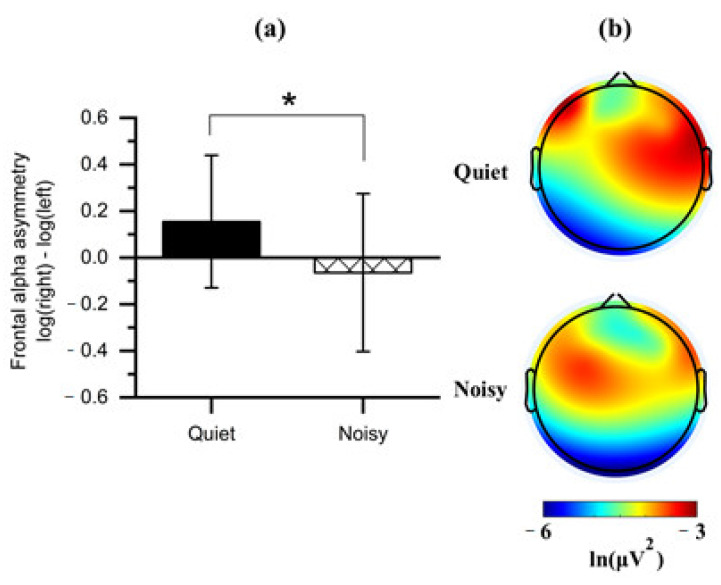
(**a**) Mean of frontal alpha asymmetry scores obtained during the quiet and noisy workplaces. Error bars indicate the standard deviations; (**b**) Topographical maps of alpha power for each workplace. Note: the alpha power is inversely associated with cortical activation. The asterisk (*) indicates *p* < 0.05.

Paired t-tests were applied to the FAA scores obtained from each workplace condition (quiet and noisy) to analyze whether work environments elicited asymmetric frontal alpha activity. Noting that the quiet workplace led to an increase in the right relative to the left absolute alpha power (*M* = 0.155, *SD* = 0.284), which was significantly differed (*t*(1, 14) = 2.408, *p* < 0.05) from the noisy workplace that showed a decreased right alpha power (*M* = −0.064, *SD* = 0.339) relative to left (Figure 6a).

We further compared all frontal regions with the two workplaces on the values of log-transformed alpha power to identify the affected frontal subregion on the FAA. Observing a significant effect on the DLPFC with the workplace conditions (Figure 6b). Four clusters of electrode channels corresponding to the DLPFC were included for further analysis.

**Figure 7:**
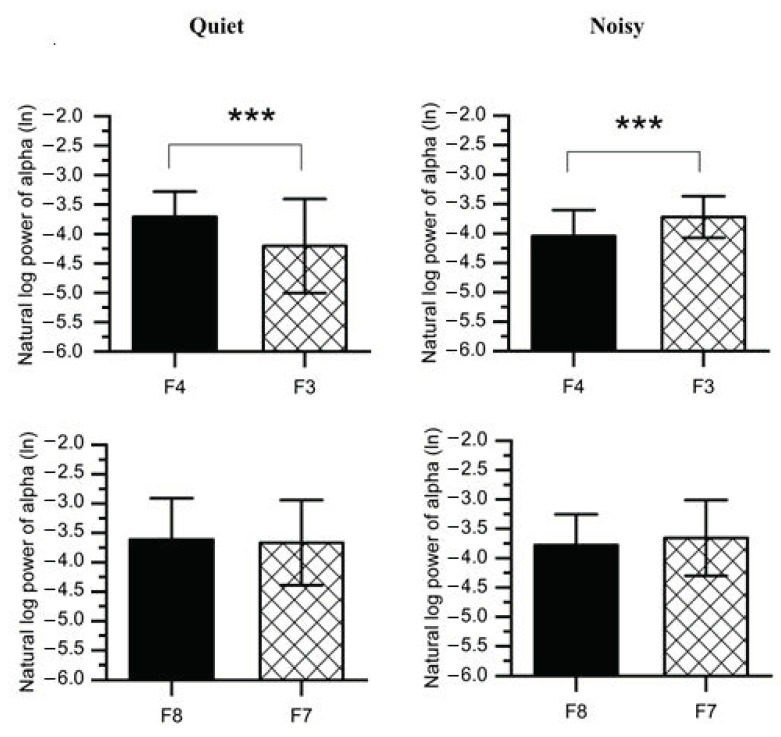
Average natural log alpha power recorded from the right frontal (F4 and F8) and left (F3 and F7) at quiet and noisy workplaces. Error bars indicate the standard deviation, and stars (***) signify significant differences *** *p* < 0.001.

To disassemble the different contributions of the alpha power collected from the right and left frontal lobes into discrepancies in the FAA scores reported for each workplace condition, paired t-tests were carried out on the values of log-transformed alpha power (Figure 7 and Table 1). The results revealed that when participants were exposed to a noisy environment, a decreased alpha power was found in the right (F4: *M* = −4.043, *SD* = 0.441; F8: *M* = −3.779, *SD* = 0.522) as compared to the left hemisphere(F3: *M* = −3.719, *SD* = 0.354; F7: *M* = −3.657, *SD* = 0.645). However, F4-F3 pair was statistically significant (*t*(1,14) = 4.535, *p* < 0.001) at noisy workplace, while no significant difference was observed between F8 and F7 (*t*(1,14) = 1.083, *p* = 0.149).

Furthermore, working at the quiet workplace elicited higher alpha power (F4: *M* = −3.708, *SD* = 0.428; F8: *M* = −3.614, *SD* = 0.705) in the right as compared to the left hemisphere (F3: *M* = −4.204, *SD* = 0.799; F7: *M* = −3.666, *SD* = 0.725). The F4-F3 comparison was also significant (*t*(1,14) = 3.202, *p* < 0.01) at quiet workplace, while the F8-F7 comparison was not significant (*t*(1,14) = 0.678, *p* = 0.254). We further compared F4-F3 and F8-F7 regions with a 2 (workplace: quiet, noisy) × 2 (location: F4-F3, F8-F7) within-subjects ANOVAs on the FAA scores (e.g., lnF4-lnF3). A significant interaction was found (*F*(2,27) = 13.213, *p* < 0.001, *η_p_*^2^ = 0.495) between workplace and location. Post-hoc pairwise comparisons with Bonferroni corrections also confirmed significant workplace effects at F4-F3 (*F*(1,28) = 23.102, *p* < 0.001, *η_p_*^2^ = 0.452) while no significant effects were found at F8-F7 (*F*(1,28) = 1.631, *p* = 0.212, *η_p_*^2^ = 0.055).

## 4. Discussion

EEG has been frequently used to evaluate the upper executive functions and mental processes. However, the underlying mechanisms applicable to EEG signals by these states are not fully clear. This study aimed to investigate the salivary alpha-amylase activity and asymmetric frontal EEG alpha power in response to the workplace environments (quiet and noisy), and their association with participants’ performance. Towards these objectives, fifteen volunteers performed the MIST at quiet and noisy workplaces while their EEG signals were recorded. We could demonstrate that workplace noise with the existence of workplace-related psychosocial stressors had a significant association with alpha-amylase level and EEG alpha power.

At the behavioral and physiological levels, participants who performed the MIST at noisy workplaces exhibited higher sAA levels than those at quiet workplaces, indicating a higher level of induced-stress at noisy workplaces (Figure 3). In support of this finding, previous studies revealed the association between high sAA levels with higher stress [46,47,48]. Besides, participants showed a declined performance in response to noisy workplaces (Figure 4), which may be owing to an increase in stress. The current study also demonstrated the dissociation of the PFC concerning workplace environments. Relatively low alpha power was observed in the PFC of all participants during the stress session at the noisy workplace as compared to the quiet workplace. These observations are due to an increase in mental stress as validated by sAA levels and consistent with findings from earlier studies [30,49,50,51]. Implying that noisy workplace-related stress exposure adversely restricts attention allocation, resulting in interference from irrelevant stimuli. These include environmental noise stressors that may impede selective attention [52,53]. Additionally, empirical studies reported that environmental noise could lead to a release of stress hormones [54].

To the best of our knowledge, this study is one of the few studies that investigated stress effects and the first to evaluate the FAA during concurrent environmental and psychosocial stressors interaction. Previous stress studies were limited to an individual stressor, ignoring combined workplace stressors as occur in the real-life workplace [55,56]. Here, we found a shift in the cortical activity associated with stress conditions in the right frontal lobe (including all electrodes) at the noisy workplace (Figure 6a). Further, our analyses categorized the frontal cortex into four regions involving mid-frontal placed electrodes (F4-F3) and more laterally electrodes (F8-F7). Interestingly, these two locations led to different interpretations. We found a significant shift in activity in the right anterior superior area (region, F4) occurs in participants exposed to the noisy workplace during stress sessions. No significant shift was found in the right anterior inferior area (region, F8) (Figure 7). Consistently, we also observed a significant shift in F4-F3 activity when we limited the interpretation to FAA_F4-F3_ and FAA_F8-F7_.

In line with the findings reported above, previous studies also indicated the significance of F4-F3 regions in examining frontal alpha activity during stress conditions [50,56,57]. The changes in the right and left DLPFC activity of participants working in different work environments may also be related to emotional regulation. The right DLPFC is associated with threat-related stress, while the left DLPFC is related to cognitive control and down-regulation of stress [58,59]. More specifically, the slight increase in the left PFC (region, F3) of the quiet workplace group may indicate the down-regulation of the stress response caused by the MIST. In contrast, the remarkable increase in the right PFC (region, F4) of the noisy workplace group may be related to the increased stress caused by noise stressors. Recent studies provided support for this conclusion through musical emotions, showing increased activity in the left and right frontal regions when listening to neutral and unpleasant music, respectively [60,61]. We could demonstrate that exposure to uncontrollable workplace noise could reflect a negative impact on participants and lead to an increased level of stress.

## 5. Conclusions

In this study, the effect of workplace noise on FAA measures and sAA activity during mental stress conditions is accessed. The results showed that workplace noise and related stress could negatively impact the cognitive functioning of users. This effect was associated with increased cortical activation in the right PFC regions, particularly in the right anterior superior area (region, F4), in comparison with the left PFC. Furthermore, the results exhibited a significant increase in sAA activity in response to workplace noise, confirming the presence of stress. This indicates that the FAA may give greater insight into neural mechanisms underlying stress effects and downregulation. Accordingly, the FAA can be used as a biomarker of workplace-related stress assessment.

Planned future work involves a further assessment of the specific link between EEG fluctuations, psychological stress responses, and various levels of noise exposure to dissociate the responses of brain sub-regions to the workplace induced stress. We anticipate that further understanding of stress effects will make an impact on the implementation of future workplace design, where the workplace will be more ergonomic to the users.

## Figures and Tables

**Table 1:** Means, standard deviations, *t*-values, and *p*-values of right (F4 and F8) and left (F3 and F7) frontal hemispheres at quiet and noisy workplaces.

Workplace	Location	Mean (*M*)	Standard Deviation (*SD*)	t-Value	*p*-Value
Quiet	F4	−3.708	0.428	3.202	0.003 **
	F3	−4.204	0.799		
	F8	−3.614	0.705	0.678	0.254
	F7	−3.666	0.725		
Noisy	F4	−4.043	0.441	4.535	0.000 ***
	F3	−3.719	0.354		
	F8	−3.779	0.522	1.083	0.149
	F7	−3.657	0.645		

Note. *t*-test: ** *p* < 0.01, and *** *p* < 0.001.

## Data Availability

The data presented in this study are available on request from the corresponding author.
